# The NYMERIA Study: A Real-World, Multicentre Contemporary Assessment of Disease- and Patient-Related Burden and Treatment Strategies in Patients with Systemic Lupus Erythematosus

**DOI:** 10.31138/mjr.160524.tns

**Published:** 2024-06-30

**Authors:** George Bertsias, Antonis Fanouriakis, Marina Bartsakoulia, Petros Galanakis, Dimitrios T. Boumpas

**Affiliations:** 1 Rheumatology and Clinical Immunology, University of Crete Medical School, Heraklion, Greece,; 2 Division of Immunity, Institute of Molecular Biology and Biotechnology-Foundation for Research and Technology – Hellas (FORTH), Heraklion, Greece,; 3 Rheumatology and Clinical Immunology Unit, “Attikon” University Hospital, National and Kapodistrian University of Athens, Athens, Greece,; 4 Medical Department Respiratory & Immunology AstraZeneca, Athens, Greece,; 5 Rheumatology and Clinical Immunology Unit, “Attikon” University Hospital, National and Kapodistrian University of Athens, Athens, Greece,; 6 Laboratory of Autoimmunity and Inflammation, Biomedical Research Foundation of the Academy of Athens, Athens, Greece,; 7 Joint Academic Rheumatology Program, Medical School, National and Kapodistrian University of Athens, Greece, Medical School, University of Cyprus, Nicosia, Cyprus

**Keywords:** activity, organ damage, biological agents, health-related quality of life, fatigue, work productivity

## Abstract

Systemic lupus erythematosus (SLE) has a spectrum of phenotypes. Its management should target remission or low disease activity, prevention of relapses and organ damage, minimisation of drug-related harms, and optimisation of health-related quality of life. Advances in our understanding of the disease pathophysiology have expanded the treatment armamentarium with targeted biologics that demonstrate superiority over conventional drugs in controlling activity, reducing flares and glucocorticoid exposure, and improving patient-related outcomes. In view of this, there is a critical need for real-world evidence providing insight into the spectrum of activity, the treatment landscape, and unmet needs among SLE patients. Such information can support regulatory and reimbursement decision-making. The primary objective of the NYMERIA multicentre study is to generate real-world evidence on the activity state of SLE patients treated in routine care settings in Greece. The overarching aim is to capture the disease burden based on both clinical aspects and the patient perspective.

## INTRODUCTION

Systemic lupus erythematosus (SLE) is a chronic multi-system autoimmune disease, with a mosaic of phenotypes spanning from mild musculoskeletal and mucocutaneous manifestations to life-threatening organ involvement. While SLE frequency shows worldwide variability attributed to genetic, environmental exposure, diagnostic and methodological differences,^1^ prevalence estimates in Greece range between 39.5 to 123.4 per 100,000 adults.^2–4^

Despite advances in the disease management, significant challenges still remain.^5^ Thus, lupus patients tend to follow a fluctuating pattern of disease activity, with periods of remission alternating with flares.^6^ Moreover, a sizeable number of individuals may experience periods with persistently active, mild-to-moderate activity. Active disease, typically requiring extended intake of glucocorticoids, carries an increased risk for adverse consequences especially accrual of irreversible organ damage, which is a major driver for excessive morbidity and mortality. Furthermore, SLE patients suffer from impaired health-related quality of life (HRQoL), comparable to their counterparts with diabetes mellitus, myocardial infarction and other chronic disorders.^7^ In fact, HRQoL in SLE shows only partial correlation with disease activity^8^; rather, it may be affected by other factors including long-standing complications (i.e., damage) and fatigue. Indeed, the latter parameter is often under-treated by the physicians due to its subjective nature and multidimensional causes despite the fact it can impact on patient’s work ability and productivity, thus resulting in increased rates of unemployment and absenteeism.^9^

It is widely recognised that SLE treatment should aim at sufficient control of disease activity under no or minimal dose of glucocorticoids.^10^ Accumulating evidence supports that sustained attainment of either remission or low disease activity – both states requiring a maintenance dose of oral glucocorticoids ≤5 and ≤7.5 mg/day of prednisone equivalent – are linked to slowing or prevention of SLE exacerbations and organ damage.^11^ The favourable effects of achieving these targets may extent to other relevant outcomes including improvements in HRQoL.^12,13^ The importance of ‘treating-to-target’ strategy has been appreciated in the 2019^14,15^ and 2023^16^ update of the EULAR (European Alliance of Associations for Rheumatology) recommendations for the management of SLE. In this context, novel biological agents approved for lupus including belimumab and anifrolumab, have demonstrated superiority over conventional drugs in achieving low disease activity/remission,^17–19^ reducing flares,^20,21^ and cumulative exposure to glucocorticoids22,23 while conferring beneficial effect on patient-reported outcomes.^24,25^ To this end, EULAR has emphasised the timely initiation of these biologics in SLE patients who despite treatment with antimalarials (with or without glucocorticoids and/or immunosuppressants), they do not achieve sufficient disease control or require >5 mg/day of prednisone equivalent.^16^

In view of the above and in light of the new targeted therapies becoming available, there is a need for contemporary, real-world evidence spanning different geographical regions and settings. This data could offer valuable insights into the spectrum and burden of disease activity, the prevailing treatment landscape, and unmet needs among individuals with SLE. Also, to aid towards a holistic patient-centred approach to the disease while also, serving as valuable input for health technology assessment submissions and supporting regulatory and reimbursement decision-making for the aforementioned awaited new technologies.^26,27^ Here, we outline the design and methodology of a multicentre, cross-sectional study employing both chart-based assessment and single visit evaluation in 200 Greek SLE patients managed under real-life condition.

## METHODOLOGY

### Overview of the study design

This is a single-country, multicentre, cross-sectional, and retrospective chart review study with a single-visit data collection schedule, which aims at including a representative sample of adult outpatients with SLE treated by rheumatology specialists in routine clinical care settings in Greece. The study will be carried out by 12 sites comprising hospital clinics experienced in SLE care in different parts of the country. No changes to the standard of care will be required and every part of patient care and clinical management will follow local medical practices and relevant national regulations, and will be decided by the participating physicians.

The conduct of this study will adhere to the applicable national regulatory requirements governing the conduct of such type of clinical research. Moreover, the study has been designed and will be conducted and reported in accordance with the ethical principles laid down in the Declaration of Helsinki, the Guidelines for Good Pharmacoepidemiology Practice (GPP) of the International Society for Pharmacoepidemiology, the STROBE (Strengthening the Reporting of Observational Studies in Epidemiology) guidelines where applicable, the EU General Data Protection Regulation (GDPR), and the local rules and regulations. The study will be completed in a single patient visit taking place within the normal clinical practice setting, and study-related information will be collected through routine clinical assessments that will be performed at the study visit, patient self-report and self-administered patient-reported outcomes (PROs), as well as through retrospective medical chart review. A representative of the Hellenic Federation of Associations of Patients, Parents, Guardians and Friends of Children with Rheumatic Diseases, “REUMAZIN”, was requested by the study team to provide feedback on suggested PROs relevant to SLE patients.

### Data sources and instruments

Data will be collected using a specific web-based data capture (WBDC) system (electronic Case Report Form [eCRF]) produced by the designated Clinical Research Organisation (CRO), which will adhere to all applicable data protection regulations and requirements. Data collection will be carried out by the participating physicians as part of standard clinical practice during routine assessments conducted at the single study visit. Additionally, patients will contribute data through the use of Patient-Reported Outcomes (PROs) and self-reports, where applicable. No further clinical, laboratory and imaging assessments are required apart from those performed as per the treating physician’s routine medical practice.

Patient source data pertaining to medical and SLE-related history, flare history, hospital-based HCRU over the past 12 months, results of laboratory tests of interest, and SLE management practices will be abstracted from patients’ medicals records and patient self-report. Current and past medications administered for SLE will be recorded and presented in terms of pharmacological categories (i.e., antimalarials, glucocorticoids [GCs], immunosuppressive, biological agents, non-steroidal anti-inflammatory drugs, other) and drug classes except for GCs, in which case active substance, route of administration and dosage (for the current GCs as well as for those received over the past 12 months before enrolment) will be also collected to allow for conversion to prednisone equivalents. Recorded terms of medical history and comorbidities will be coded using the Medical Dictionary for Regulatory Activities (MedDRA) terminology (the last updated version available at the onset of medical coding) in preferred term and system organ class.

SLE clinical instruments will include: a) the clinical SLEDAI-2K (cSLEDAI-2K),^28^ in which the immunological items (anti-dsDNA and complement levels) are excluded, thus facilitating the evaluation of disease activity in daily clinical practice when results from serological tests are often lacking; b) clinical SLEDAI-2KG, which represents a modification of the SLEDAI-2K introduced to describe disease activity while accounting for GC dose category^29^; c) Physician Global Assessment (PhGA), which is a responsive instrument that reflects the clinician’s judgment of overall SLE disease activity on a scale between 0 (no), 1 (mild), 2 (moderate) and 3 (most severe disease activity/severity)^30^; and d) the SLICC/ACR Damage Index (SDI), which has been extensively validated to capture irreversible dysfunction/damage in 12 organ/systems accrued as a consequence of either disease activity, received treatments or comorbidities.^31^

The following PROs will be collected via self-administered paper questionnaires completed by the patients themselves at study enrolment: a) LupusPRO, which is a validated survey tool developed for lupus patients to measure the impact of the disease and its treatment both on their health-related (HRQoL) and non-health-related quality of life (N-HRQoL)^32^; b) EQ-5D-5L,^33^ a generic HRQoL instrument that produces country-specific value sets therefore facilitating the calculation of quality-adjusted life years that are used to inform economic evaluations of health care; c) Work Productivity and Activity Impairment (WPAI):Lupus,^34^ which is a validated self-administered Lupus-specific version of the WPAI questionnaire that records impairment in work and general activities due to SLE over a 7-day period; and d) Fatigue Severity Scale (FSS), a self-administered 9-item scale measuring the severity of fatigue and its effect on patients’ daily functioning.^35^ Self-administered Greek versions of the aforementioned PROs will be provided to the enrolled patients. The questionnaires will be distributed prior to the patient undergoing any additional procedures or clinical examinations during the study visit. This will occur before the patient receives information about the current state of their disease or any adjustments to the therapeutic strategy. This timing is deliberate to ensure that interactions with healthcare professionals do not unduly impact the patient’s responses.

### Study population, inclusion and exclusion criteria

A total of 200 patients eligible for participation in the study according to the inclusion and exclusion criteria outlined below will be recruited over a 12-month period. Each participating site is expected to recruit between 10 and 30 patients. Patient selection will be based on consecutive sampling method, i.e., all consecutive eligible and consenting patients are expected to be included in the study. To mitigate potential selection bias, physicians will be requested to consecutively enrol the first eligible patients (based on the site-specific target) attending their clinic/office over the pre-specified study recruitment period.

For inclusion in the study, patients should fulfil the following criteria: i) male or female adult outpatients aged between 18 and 65 years old (inclusive); ii) documented, physician-based diagnosis of SLE ascertained by the formal classification criteria for SLE (1982/1997 ACR and/or 2012 SLICC and/or 2019 EULAR/ACR criteria); iii) disease duration of at least 12 months; iv) information available for scoring the cSLEDAI-2K over the 30 days prior to enrolment, or for patients whose physicians plan to perform such measurements at the study visit as part of their routine practice, regardless of their decision to include the patient in the current study.; v) accurate and complete medical records for data abstraction to meet the objectives of the study; vi) patients willing and able to read, understand and complete the provided patient questionnaires; vii) patients must provide a written informed consent prior to inclusion in the study.

Patients will be excluded if any of the following criteria are met: i) coexisting systemic rheumatic/autoimmune disease (including, among other others, rheumatoid arthritis, multiple sclerosis, systemic sclerosis); b) active malignancy diagnosed prior to SLE diagnosis; c) ongoing pregnancy; d) treatment with any non-approved drug/device/intervention or patients who have received any investigational product within 1 month or 5 half-lives of this product (whichever is longer) at the time of enrolment; e) patients who have already been enrolled in this study or in a study of the same design which the physician has good reasons to believe that it is this study (to exclude enrolment of the same patient by two different sites).

### Study outcomes and statistical analysis

The primary and secondary outcomes of the study are outlined in **Table 1**. Statistical analysis and generation of tables and patient data listings will be performed using the latest version of SAS^®^ software available at analysis onset. Statistical analysis will descriptive, not focusing to confirm or reject any pre-defined hypotheses. Continuous variables will be summarised using descriptive statistical measures [number of patients with available observations (npt), number of missing observations (nmiss), mean, SD, median, 25th and 75th percentiles, minimum (min) and maximum (max)] and categorical variables will be displayed using frequency tables including the respective 95% Clopper-Pearson Confidence Intervals (CIs) (where indicated), in the overall study population and the relevant defined subgroups by disease activity state. The normality of distribution of continuous variables will be examined using the Shapiro-Wilk test.

**Table 1.** Primary and secondary objectives of the NYMERIA observational study.

**Primary Outcomes**		
[1]	-	Proportion of patients classified as having at least moderate clinical disease activity, defined by a cSLEDAI-2K score ≥6.
	-	Proportion of patients classified as having at least moderate clinical disease activity, defined by a cSLEDAI-2KG score ≥6.
	-	Proportion of patients classified as having at least moderate clinical disease activity, defined by a PhGA score >1.
[2]	-	Frequencies of different clinical disease activity states (i.e., no activity, mild, moderate, high, and very high activity) using the cSLEDAI-2K, the cSLEDAI-2KG, and the PhGA score, respectively.
	-	Reclassification rate and concordance (kappa coefficient) between the different indices (i.e., cSLEDAI-2K and cSLEDAI-2KG; cSLEDAI-2K and PhGA; and cSLEDAI-2KG and PhGA) in terms of patient distribution in the “inactive/mild” and the “at least moderate” disease activity state.
[3]	-	Proportion of patients in clinical remission (as per the 2021 DORIS definition) defined as cSLEDAI-2K=0 and PhGA<0.5; the patient may be on AMs, low dose GCs (prednisolone equivalent<5 mg/day), and/or stable IS including biologics.

**Table:** Secondary Outcomes^1^

[1]	-	Patient’s demographic and clinical characteristics, overall and by disease activity state.
[2]	-	LupusPRO overall score, total HRQoL and N-HRQoL constructs’ scores, and individual domain and item scores at the study visit.
	-	EQ-5D-5L utility index score and EQ-Visual Analogue Scale (VAS) score at the study visit.
	-	Proportion of patients with reported problems for each level on each dimension of the EQ-5D and proportion of patients with ‘no problems’ (i.e., level 1) and ‘with problems’ (i.e., level 2 to 5).
[3]	-	WPAI:RS domain scores referring to absenteeism, presenteeism, and work productivity loss, among the study population who are employed at the study visit.
	-	WPAI:RS domain score referring to activity impairment among all patients.
[4]	-	FSS total and item scores.
	-	Proportion of patients with an FSS total score ≥36 indicating the presence of clinically significant fatigue.
[5]	-	Total SDI score.
	-	Proportions of patients with and without organ damage (i.e., with SDI score ≥1 and 0, respectively), and frequencies of the different SDI states (SDI score: 0, 1, 2, 3, 4 and ≥5).
	-	Frequency of damage in the different organ systems/domains and items of the SDI.
[6]	-	Number of swollen (SJC) and of tender joints (TJC) based on 28-joint counts, and total number of affected joints (swollen and/or tender).
	-	Moderate to severe joint disease activity (active joint count of ≥8 tender and/or swollen joints).
	-	Swollen to tender joint ratio (STR [SJC/TJC]) and frequencies of low (STR <0.5), moderate (0.5 ≤ STR ≤ 1.0), and high (STR >1.0) joint disease activity.
	-	Percentage of body surface area (BSA) affected by SLE.
	-	Moderate to severe skin disease as reflected by a BSA affected by SLE ≥10%.
[7]	-	Frequencies of current pharmacologic treatments by type/pharmacological category [monotherapy, combination regimen; AMs, GCs, IS, biological agents, NSAIDs, other], drug class, treatment duration, and number of dose intensifications since the start of current treatment including reason for intensification.
	-	Types and frequencies of past (before the start of current treatment) pharmacologic treatment modalities by pharmacological category and drug class.
[8]	-	Correlation coefficient and association (through linear regression analysis) of the SLEDAI-2K score, SLEDAI-2KG score, and PhGA score (as continuous variables), each with the following: SDI score; LupusPRO HRQoL total and individual domain scores; LupusPRO N-HRQoL total and individual domain scores; WPAI overall work productivity loss; WPAI activity impairment score; FSS score.
[9]	-	Association of patient, disease and treatment characteristics with the presence of at least moderate disease activity determined using the cSLEDAI-2K.
	-	Association of patient, disease and treatment characteristics with the presence of at least moderate disease activity determined using the cSLEDAI-2KG.

1 The numbering of the outcomes follows the numbering of the corresponding objectives. All outcomes will be assessed in the overall study population and in the subpopulations 1A / 1B & 2A / 2B, except for #8 and #9.

The concordance between the different indices will be determined using the kappa coefficient and the percentage of agreement. The correlation and the association of the cSLEDAI-2K, SLEDAI-2KG, and PhGA scores with the SDI and selected PRO scores will be examined using the Pearson’s or Spearman’s correlation coefficient and linear regression models, respectively. Logistic regression models, using both the univariable and multivariable approach, will also be used to evaluate the association of patient, disease and treatment characteristics with the presence of at least moderate disease activity, as determined by cSLEDAI-2K and by cSLEDAI-2KG, in the overall study population. In the context of the exploratory objectives, the annual rate of flares, hospitalisations and ED visits with the respective 95% Poisson confidence intervals (CIs) will be estimated. Missing data will be reported. No imputation methods will be performed in regards to missing data with the exception of partial dates which will be defined in more detail in the SAP. With regards to the scoring and handling rules of any missing PRO items, the relevant scoring instructions, where available, will be followed. All statistical tests will be two-sided and performed at a 0.05 significance level.

### Methods to minimise bias

To lessen confounding due to the potential of patient selection bias, a consecutive sampling process will be utilised. Physicians will be requested to provide documentation of the screening process results for both consented and non-consented patients. Patient information/recall bias during collection of PRO data will be avoided by the use of widely used tools that rely on a relatively short-term (i.e., one week for FSS and WPAI:Lupus, and 4 weeks and 3 months for the LupusPRO) or no recall period (for the EQ-5D-5L). Additionally, patients should complete the self-administered PROs on their own before any study-specific procedures or clinical assessments are performed, and before they are informed about their disease status or any changes to their current treatment (where applicable). This approach is intended to prevent any response bias.

### Sample size

In view of the exploratory/descriptive nature of the study and based on practical considerations, a sample size of 200 eligible patients is considered both feasible and sufficient for estimating the primary outcome measures. This number offers a maximum margin of error of 7.1% (with 95% CI using the Clopper-Pearson exact method ranging between 42.9% and 57.1%) for the estimation of a percentage of 50%, which represents a scientifically acceptable level of precision. Also, this sample size ensures that for any of the defined study subpopulations representing at least 50% of the overall population, frequency estimates of interest may be determined with a margin of error not exceeding 10.2%, therefore ensuring sufficient precision to draw meaningful conclusions.

## CONDUCT OF THE STUDY AND REGULATORY ASPECTS

Before the first subject is enrolled in this study and any study-related procedures are undertaken the following should be fulfilled: a) written approval of the study by the Ethics Committee/Institutional Review Boards of the participating Hospitals, according to local regulations; and b) agreements between the sponsor (AstraZeneca) and each Investigator/Institution are signed. Upon the enrolment of each subject into the study, the participating physician shall review of patient’s medical history and current treatment, in order to confirm if eligibility criteria are met, explain the aims of the study to the patient, obtain signed informed consent, followed by collection of the data through routine clinical assessments, medical chart review, patient self-report and self-administered PROs, and completion of the electronic case report forms (eCRFs).

A subject will be withdrawn from data collection within the study in case of patient’s withdrawal of informed (at any time and for any reason) or erroneous enrolment (i.e., the subject does not meet the eligibility criteria for participation in the study).

The study will use eCRFs provided by the Sponsor through a dedicated system. The eCRF system, designed by a qualified contract research organisation (CRO), adheres to data protection regulations. Patients will be identified by unique numbers. Investigators or authorised personnel will enter data following the Investigator Instructions Manual. Data will be immediately saved and tracked for changes. Upon completion, the eCRF will be electronically signed by the Investigator, and a copy will be archived on-site. Quality control mechanisms will ensure data integrity, outlined in the study-specific monitoring, data management, validation plans, and SAP. Real-time edit checks will handle discrepancies, and source data verification during monitoring visits will mini-mise transcription errors. Statistical analysis and Clinical Study Report (CSR) development will be performed by a designated CRO. Before final database lock, a comprehensive SAP will be drafted, including statistical software and data imputation methods. Electronic archives of all statistical programming and related documents will be maintained. e-CRF archival for each site will be distributed on CD-ROMs or USB sticks after study completion. The designated CRO will review eCRF data for completeness and accuracy, following the DVP. Medical plausibility checks will be conducted by the Sponsor’s personnel. Monitoring, telephone contacts, and on-site visits will ensure study oversight.

## DISCUSSION

Herein, we present the rationale and methodology of a multicentre observational study in Greece, which aims to capture the range and impact of disease activity and severity, treatment patterns, and areas of need that exist among individuals with SLE. Previously, a nationwide study of 381 Greek SLE patients followed in seven hospital clinics had reported a high frequency of chronic active (11.6%) and relapsing-remitting (60.6%) disease patterns, as well as of usage of glucocorticoids (66.0%, 22.0% at a dose exceeding 7.5 mg/day prednisone equivalent) and immunosuppressive or disease-modifying antirheumatic agents (97%).^36^ Of note, the aforementioned study was enriched in patients with active disease (ascertained by change or intensification in therapy) and abnormal lupus serology (increased anti-dsDNA autoantibodies and/or low serum C3/C4 levels). Due to its explicit design and inclusion criteria, the NYMERIA study seeks to obtain more generalisable, contemporary estimates of the disease activity and severity of SLE patients managed in Greece, the use of various treatment modalities (including biologics) while also, addressing the patient perspective through relevant PROs.

Collecting data on real-world management practices and their outcomes help to bridge the knowledge gap between clinical research which is conducted in the strict environment of controlled randomised settings and that performed in daily clinical practice. The latter follow less restrictive methodological standards regarding patient selection, treatment, and other design aspects. Consequently, their results are often more generalisable, particularly for populations with complex diseases like SLE. Their cross-sectional aspect can provide a snapshot-based approach to document prevalence rates and other outcomes of interest for subgroups of population at a given timepoint in a routine clinical practice setting, which can be used on the planning of health services or the determination of healthcare practices, while being less resource-demanding and less time-consuming compared to other study designs. To this end, and despite variations in the methodology and reported outcomes, observational studies from Europe and the United States have been valuable in demonstrating that insufficiently controlled disease is not uncommon in SLE therefore, highlighting the need for novel, more effective treatment modalities and strategies.^37–42^

In SLE, permanent organ damage (quantified by the SDI) has emerged as a powerful determinant of multiple adverse patient outcomes including excessive morbidity and healthcare utilisation, impaired HRQoL, and premature death.^36,43^ Consequently, prevention of damage accrual has been recognised as a distinct therapeutic goal in SLE, which can be facilitated by lowering disease activity, minimising exposure to glucocorticoids and controlling comorbidities.^44^ In agreement with worldwide estimates, earlier findings from single centres in Greece have suggested that organ damage may accumulate at a rate of 18% within the first 6 months following SLE diagnosis,^45^ with this figure rising to 32% after an average disease duration of 7 years.^4^ The current study will provide updated rates of damage in a representative group of SLE patients managed at real-life setting, with the possibility to stratify results according to pertinent demographic and disease characteristics.

There is often discordance between physicians and SLE patients in terms of the activity and status of the disease,^46^ which denotes the necessity that patient perspectives are also taken into consideration especially in the context of shared decision-making process.^16^ Notably, the unpredictable nature of SLE, along with its physical and emotional impact, may significantly impact the overall well-being and quality of life for individuals with SLE.^47^ Furthermore, a complex interplay has been reported between disease-intrinsic characteristics (e.g., type and severity of manifestations), organ damage, treatment-related harms, pain, fatigue, psychosocial factors, and mental disorders with HRQoL, work ability and productivity, which denotes the importance of holistic approach to these patients.^47–52^ Our research marks a significant milestone as it unveils novel insights into patient-reported measures, including HRQoL, fatigue, and work productivity among SLE patients managed in Greece. Results will shed light on these crucial aspects within the country context, further enhancing our understanding of the impact of SLE on patients’ daily lives. The study faces potential errors due to incomplete or inaccurate patient record data, leading to hidden biases. Efforts to mitigate this include requiring sufficient medical records for study eligibility and setting a threshold for missing data in multivariable models. If a subpopulation falls below 100 patients, there may be a significant margin of error, thus affecting the validity of results. Nonetheless, any real-world evidence generated outside the study’s primary aim can be valuable. The observational nature of the study may introduce confounding factors, which will be analysed through appropriate multivariable analyses. Unmeasured confounding will be qualitatively discussed in the results. Internal validity is maintained through source data verification and quality assurance. Generalisability is enhanced by enrolling patients from diverse locations in Greece, though non-probability sampling makes the study’s overall generalisability less robust. Despite these limitations, the value of such studies lies in their ability to provide real-world evidence by capturing data generated during routine clinical care. This makes them more representative of the study population and the observed outcomes compared to randomised controlled trials, which offer information on a select group of patients chosen based on strict and narrow eligibility criteria and treated in a controlled environment.

In conclusion, the NYMERIA multicentre study aspires to contribute to our knowledge of the disease burden and unmet needs among Greek SLE patients by assessing objective disease indices, the usage of therapies and a number of validated PROs. This collaborative and multi-faceted approach could help foster a more nuanced and tailored approach to addressing the challenges faced by SLE patients and healthcare providers alike.

## SUPPLEMENTARY MATERIAL

### List of Study Sites

- Rheumatology and Clinical Immunology, University of Crete Medical School, Heraklion, Greece
- Rheumatology and Clinical Immunology Unit, “Attikon” University Hospital, National and Kapodistrian University of Athens, Athens, Greece
- Rheumatology Unit, First Department of Propaedeutic Internal Medicine, Medical School, National and Kapodistrian University of Athens, Joint Rheumatology Program, Laiko Hospital, Athens, Greece
- Department of Pathophysiology, School of Medicine, National and Kapodistrian University of Athens, Athens, Greece
- Department of Rheumatology, “G. Gennimatas” General Hospital, Athens, Greece
- Clinical Immunology-Rheumatology Unit, 2 Department of Medicine and Laboratory, National and Kapodistrian University of Athens, Athens, Greece
- Rheumatology Clinic, Department of Internal Medicine, Medical School, University of Ioannina, Ioannina, Greece
- Department of Rheumatology and Clinical Immunology, Faculty of Medicine, School of Health Sciences, University General Hospital of Larissa, University of Thessaly, 41110, Larissa, Greece
- Division of Rheumatology, Department of Internal Medicine, Patras University Hospital, University of Patras Medical School, Patras, Greece
- Department of Rheumatology, Evaggelismos General Hospital, Athens, Greece
